# Possibility for the Conjugated Use of Photodynamic Therapy and Electrosurgical Devices

**DOI:** 10.1371/journal.pone.0136194

**Published:** 2015-08-18

**Authors:** Francisco de Assis Martins Gomes Rego Filho, Romualdo Arthur Alencar Caldas, Cristina Kurachi, Vanderlei Salvador Bagnato, Maria Tereza de Araujo

**Affiliations:** 1 Instituto de Física, Universidade Federal de Alagoas, Maceió Alagoas, Brazil; 2 Hospital Universitário HUPAA, Universidade Federal de Alagoas, Maceió, Alagoas, Brazil; 3 Instituto de Física de São Carlos, Universidade de São Paulo, São Carlos, São Paulo, Brazil; Massachusetts General Hospital, UNITED STATES

## Abstract

Because tissue optics limits the treated volume during anti-tumor Photodynamic Therapy (PDT), its conjugation with prior tissue debulking has been suggested clinically. In this context, the conjugation of radiofrequency ablation and PDT has already been demonstrated. However, the basic principles that enable the success of these protocols have not been discussed. This proof-of-principle study analyzes the possibility of conjugating electrosurgery (ES) and PDT, analyzing different sequences of photosensitizer (PS) administration in an animal model. The animals were distributed over five groups: ES, PS+Light, PS+ES, ES+PS+Light and PS+ES+Light. The PS Photogem was administered systemically. An electrosurgical unit (480 kHz) was used to remove a portion of the liver, leaving a plane surface for PDT illumination (630 nm, 150 J/cm²). Fluorescence was collected during the stages of the experiment to monitor the PS accumulation. After 30 hours, histological processing was performed. The fluorescence spectra revealed strong Photogem emission in both administration sequences (ES+PS; PS+ES), and little PS bleach after ES was observed. The maximum necrosis depth was observed for the PS+ES+Light group—(716 ± 75) μm—higher than its respective control group (160 ± 28) μm, proving successful conjugation. Histological features from ES and PDT on both conjugation sequences were observed. Pre-photosensitized tissue presented decreased ES-related thermal damage. A simple physical hypothesis, based on the Joule effect and the tissue electrical conductivity, was proposed to support these findings. In conclusion, the results successfully demonstrated the possibility of conjugating ES and PDT in a single protocol.

## Introduction

Photodynamic Therapy (PDT) is a technique used to attack tumors and localized infections. It begins with the administration of a photosensitizer (PS), which concentrates in the tumor after a characteristic accumulation time. The optical excitation of the PS triggers a sequence of chemical and biological processes, leading to tumor death. The formed chemical species have short lifetimes, causing the local destruction of the cells, preserving the surrounding healthy tissue [1,2].

Most anti-tumor PDT protocols employ Hematoporphyrin Derivates (HpD) as photosensitizers. Among them, Photogem has presented promising results to treat different types of tumors since its appearance [3]. It is usually excited by red light (~ 630 nm). However, because of the strong light absorption and scattering of biological tissues, such penetration is limited to a few millimeters [4–6]. Therefore, PDT alone is not effective in treating bulky tumors.

Although surgery is still the most adopted procedure in such cases, it involves the removal of the tumor and a portion of surrounding healthy tissue, defined as a safety margin. To improve healthy tissue preservation, a recently growing strategy is to conjugate PDT and surgical debulking in a single protocol [7,8]. This approach guarantees safer tumor extinction and minor recurrence rates. Among all debulking techniques, Electrosurgery (ES) has the potential to be conjugated with PDT based on its properties and widespread availability [9].

Electrosurgery is based on the radiofrequency (RF) ablation of soft tissues [10]. This process causes surface modifications and thermal damage [11,12]. The surface modifications cause an increase on the diffuse reflectivity, decreasing the number of photons that couples into the tissue. The thermal damage changes the tissue optical properties, such as the scattering cross section, which increases for shorter wavelengths [13]. Moreover, the combination of ES and PDT has other theoretical limitations. Firstly, if the tissue is previously photosensitized and cut via electrosurgery, the RF current would cause the bleach of the PS molecules. Secondly, if ES were applied first, the tissue damage would decrease the drug uptake in the damaged region. Both processes will influence the PDT threshold dose [14] and decrease the PDT efficiency after ES.

Nonetheless, the literature has no records on how the electrosurgical currents would influence the tissue optics. Some review studies compare the clinical outcome of using ES and PDT as separate techniques, but not conjugated in a single procedure [15–17]. Some papers propose other techniques (CO_2_ and Er:YAG laser ablation, Microdermabrasion etc.) to promote tissue debulking prior to PDT and enhance the tumor extinction [8,10,18–26]. However, their main focus is on the treatment outcome and not on the underlying phenomenology. Furthermore, the sequence in which the PS and ES are applied can be changed before the PDT illumination. This suggests protocols with different sequences of conjugation. A recent study explores this variable when conjugating PDT with CO_2_ laser ablation in healthy liver [27]. Different histological properties were found only by changing the PS type and the conjugation sequence.

This proof-of-principle study aims to demonstrate and analyze protocols that conjugate PDT and electrosurgical excision in a single procedure. The study was performed on an animal model and analyzed for different protocol sequences. Real-time fluorescence was used to monitor the drug availability *in vivo* and also to analyze thermal damage effects after the application of RF currents. Histopathology analysis was used to distinguish between the inherent ES and PDT damage profiles and also to calculate the total necrosis depth, confirming their co-occurrence in all combination sequences. The observed results were related to the tissue physical properties, such as electrical conductivity, heat diffusion, fluorescence and light penetration.

## Materials and Methods

The animal model consisted of fifteen male Wistar rats, weighing between 350 g and 400 g. To prove the possibility of conjugating ES and PDT in response to the applied external parameters (illumination, applied current, tissue surface modifications, etc.), a model that uses healthy tissue is sufficient. The animals were obtained from the Biotério Central of the FMRP, Universidade de São Paulo, Brazil. The study was formally approved by the Internal Review Board for animal studies.

Based on a previous study [28], a monopolar electrosurgical unit (HF-120, WEM, Brazil) operating with a straight-wire delivery electrode was used. It employed a blended cut (i.e. promoting simultaneously cut and coagulation) for all experimental groups that involved ES, using fixed parameters (45 W). These parameters were carefully tested to avoid adherence and facilitate the production of a plane surface for PDT illumination. The active electrode was handled manually. Electrosurgery and PDT necroses have different damage profiles, which can be identified separately. Therefore, the co-occurrence of both damage profiles was used as a criterion to validate the success of the ES+PDT conjugation sequences. The animals were shaved at the region in contact with the return electrode and divided randomly into five groups (Table 1).

**Table 1:** Experimental groups.

Group	Short name	Description	Number of animals
**G1**	Control: ES	Electrosurgery only	N = 3
**G2**	Control: PDT	Photodynamic Therapy only	N = 3
**G3**	Control: PS+ES	PS administration followed by electrosurgical cut	N = 3
**G4**	ES+PS+Light	Electrosurgical cut followed by PS administration and posterior 630 nm illumination for PDT	N = 3
**G5**	PS+ES+Light	PS administration followed by electrosurgical cut and posterior 630 nm illumination for PDT	N = 3

**Fig 1:**
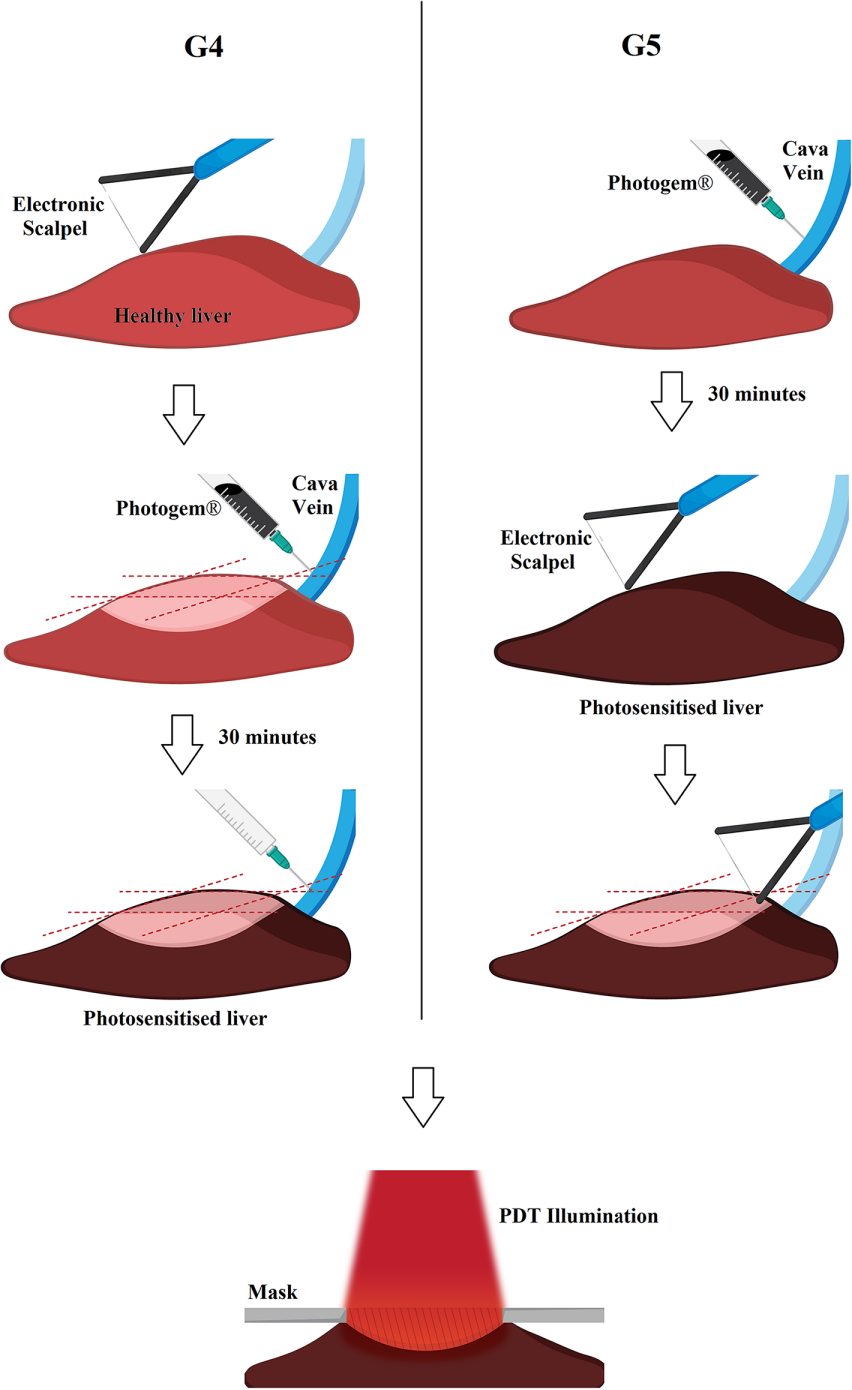
Experimental steps. The columns represent individual steps of the protocols G4 (left) and G5 (right). The light red color represents the liver before full photosensitization.

The animals were previously anesthetized to minimize suffering, using a sterile solution containing 100 mg.kg^-1^ body weight of Ketamine (Ketamine Agener 10%, União Química Farmacêutica Nacional S/A, Brazil) and 6 mg.kg^-1^ body weight of Xylasine (Coopazine, Coopers Brasil Ltda, Brazil). The liver was accessed via laparotomy procedure. Photosensitization was performed by the injection of Photogem (Moscow, Russia) into the inferior vena cava at a dose of 1.5 mg.kg^-1^ body weight. The maximum concentration of Photogem is achieved in the liver 30 minutes after its administration [29,30]. Electrosurgery and/or illumination were performed at this point in groups G2, G3 and G5. For group G4, the PS is administrated immediately after the ES cut, while illumination occurs 30 minutes after. The portion of removed tissue was less than 10% of the right lobe of the liver. The following procedures were performed on the remaining surface (Fig 1).

The PDT illumination was performed using a 630 nm CW diode laser (Eagle Heron, QuantumTech, Brazil), in an area of 1 cm^2^, delimited by an external mask. An intensity of 150 mW.cm^-2^ and exposure time of 1000 s was applied (total dose D = 150 J.cm^-2^). The beam was directed on the exposed liver surface using an optical fiber with a micro-lens at its end that generated a flattop beam profile. All animals were sacrificed 30 hours after the procedures [6] using a carbon dioxide chamber. The liver was removed to produce specimens for posterior histological processing. The slices were taken transversely to the treated surface, near the center of the lesion. In order to visualize cell/tissue structures, the conventional haematoxylin-eosin staining process was applied. Images from every slide were obtained using a microscope (Observer Z1, Carl Zeiss, Germany). Three features were analyzed with this technique: erythrocyte extravasation (hemorrhage), neutrophil infiltration and coagulation. Three (3) slides were obtained for each specimen, resulting in nine (9) slides per group, used to calculate the statistical mean damage intensities.

The total depth of necrosis was calculated to measure the treated volume that arises from the conjugation sequences. Two histological slides from every animal were used to measure the necrosis depth in 10 different depth points, resulting in sixty (60) independent depth measurements per group. The measurements were made using the AxioVision LE software (Carl Zeiss, Germany), and used to calculate statistical means and standard deviations.

At every step of the experiments an *in vivo* fluorescence spectroscopy system (Spectr-Cluster v. 2.05m, Cluster, Russia) was used as a real-time assessment tool. It operates with a 532 nm Nd:YAG laser coupled to a Y-type optical probe. The collected spectra contained residual backscattered laser light at 532 nm, which was used to normalize the emission spectra to its intensity. Next, frequencies below 540 nm were cut off and the resulting spectra were re-normalized. In order to obtain a reliable emission spectrum of the treatment region, seven (7) spectra from different points of the lesion extent were collected, for every animal, at all steps of the experiment, resulting in 21 spectra per experimental group. The spectra were used to calculate the average emission curves.

## Results

**Fig 2:**
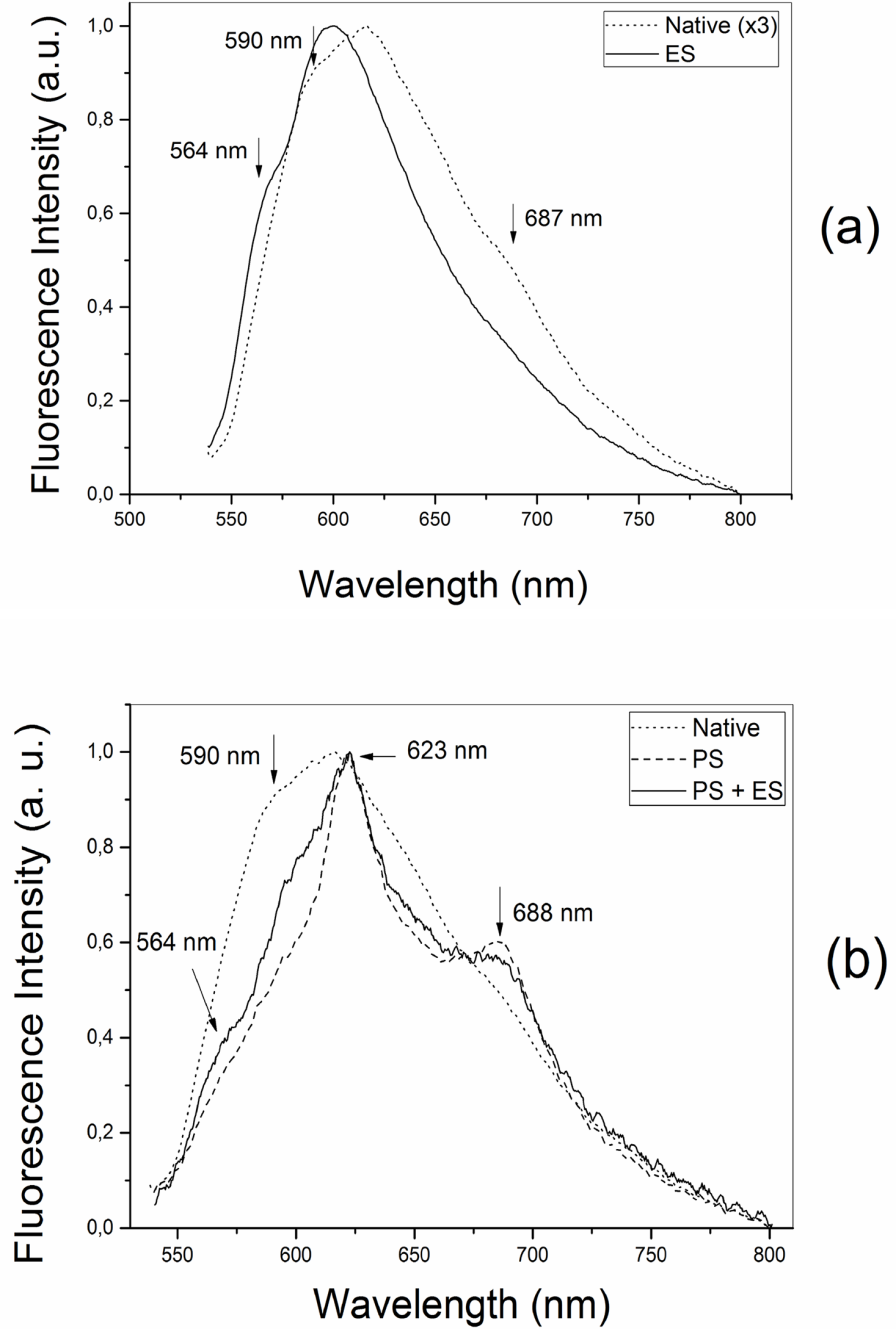
Fluorescence analysis. (a) Autofluorescence from the native liver (dotted line) and the liver tissue damaged by electrosurgery (solid line). (b) Fluorescence spectra of the pre-photosensitized liver damaged by ES (solid line), the photosensitized native liver emission (dashed line) and autofluorescence (dotted line).

**Fig 3:**
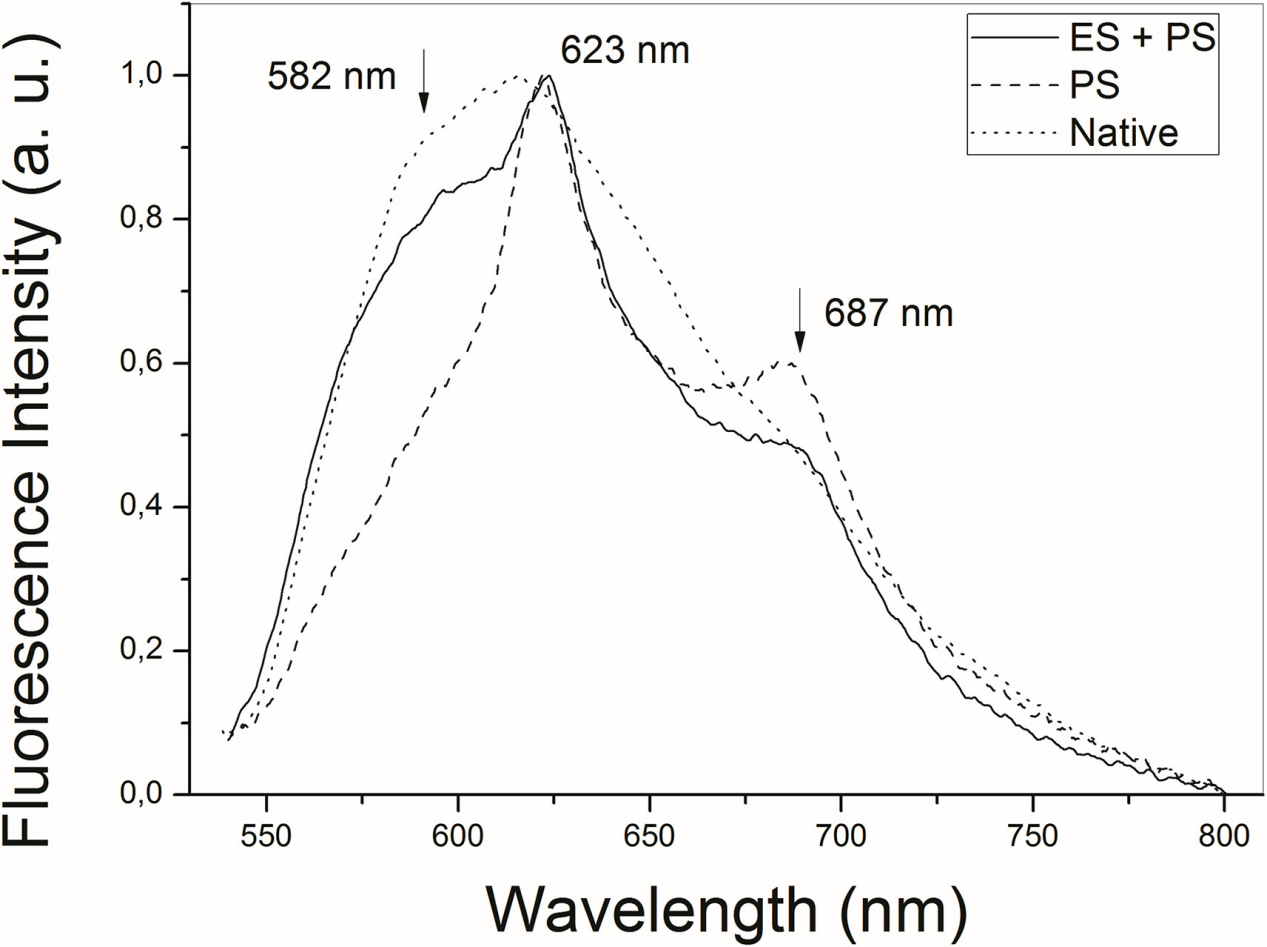
Fluorescence analysis. Fluorescence spectra of the tissue previously damaged by ES and subsequently photosensitized (solid line), versus the photosensitized only (dashed line) and native tissue (dotted line).

Real-time monitoring of the treatment site was performed by collecting the light emitted after laser excitation during the stages of the experiment. The resulting graphs are shown in Figs 2 and 3.

In Fig 2A, the fluorescence of rat livers belonging to group G1 is shown and compared to the native tissue fluorescence (autofluorescence). The normalization factor used in the second step of the normalization process is a qualitative measure of the intensity ratio. In the ES damaged tissue fluorescence this factor was of approximately 3. Consequently, ES damage caused an increase in the collected optical intensity. Spectral differences could also be observed and are indicated by arrows. The main emission peak is blue-shifted after the ES treatment (native tissue—618 nm; damaged tissue—601 nm). A similar shift occurs with the 590 nm shoulder, which appears with decreased relative intensity at 564 nm after ES. In addition, the autofluorescence presents an emission peak at 687 nm, which no longer appears after electrosurgery. A decrease in the full width at half maximum (FWHM) is observed on the tissue fluorescence damaged by electrosurgery.

Fig 2B shows the liver surface fluorescence when ES acts on pre-photosensitized tissues (group G3, solid line). The emission spectrum of the native tissue after PS administration (dashed line) and autofluorescence (dotted line) are plotted on the same figure for comparison. In the PS+ES curve (solid), an emission peak at 564 nm is evident and is related to ES damage. A shoulder near the 590 nm is observed, which is related to the native tissue unaffected by electrosurgery. In addition, two peaks are observed at 623 nm and 688 nm, which are related to the Photogem fluorescence.

For group G4, the spectra evaluated the Photogem uptake before illumination. The spectra were collected 30 minutes after the application of the PS, and the average behavior is shown in Fig 3. The Photogem emission peaks are also present in this group. However, when compared to Fig 2B, a significant shoulder on the emission curve is observed at 582 nm. In addition, the decrease on the FWHM was only observed in the photosensitized native tissue (dashed line).

**Fig 4:**
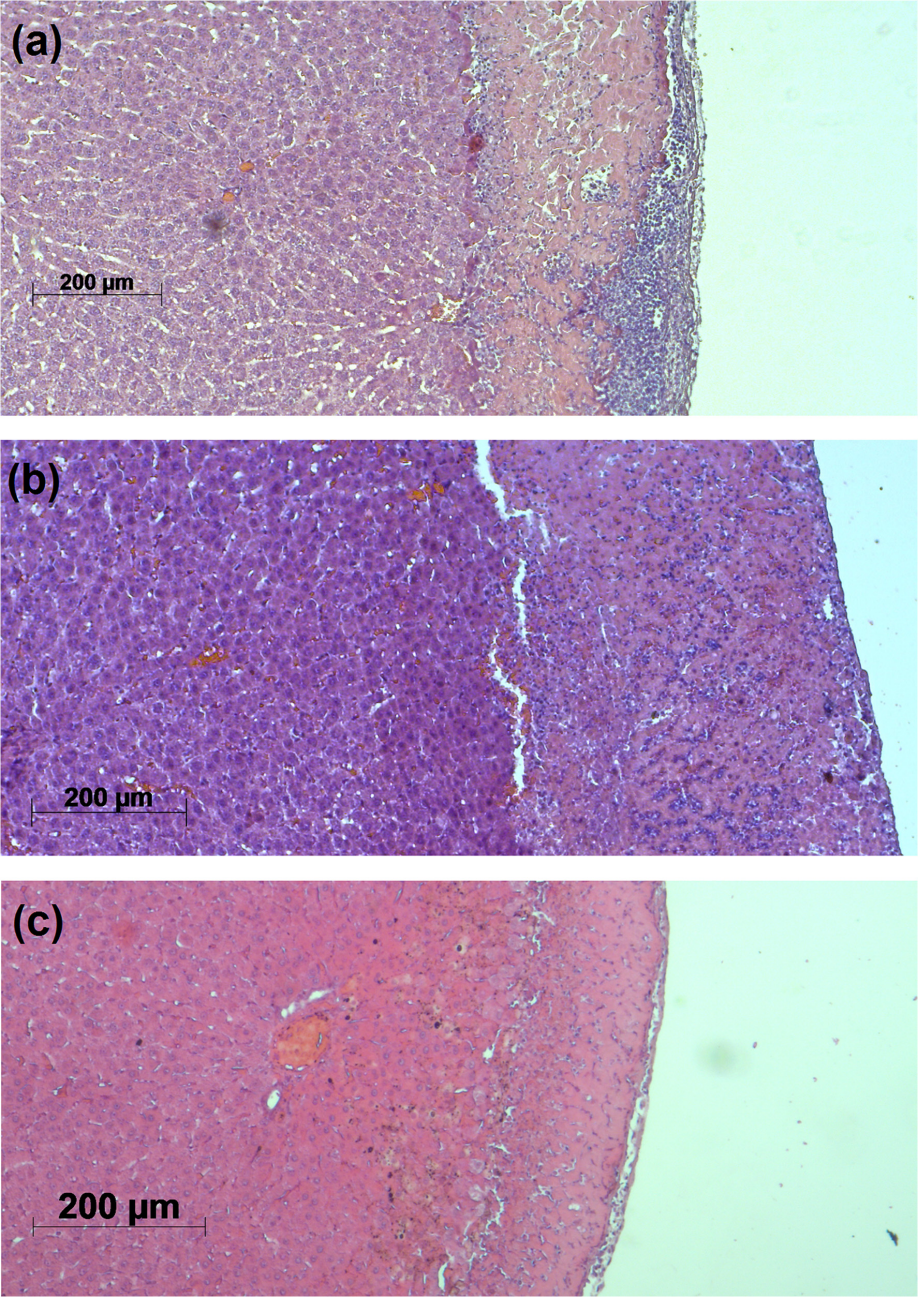
Representative histopathological slides. (a) G1; (b) G2; (c) G3.

**Fig 5:**
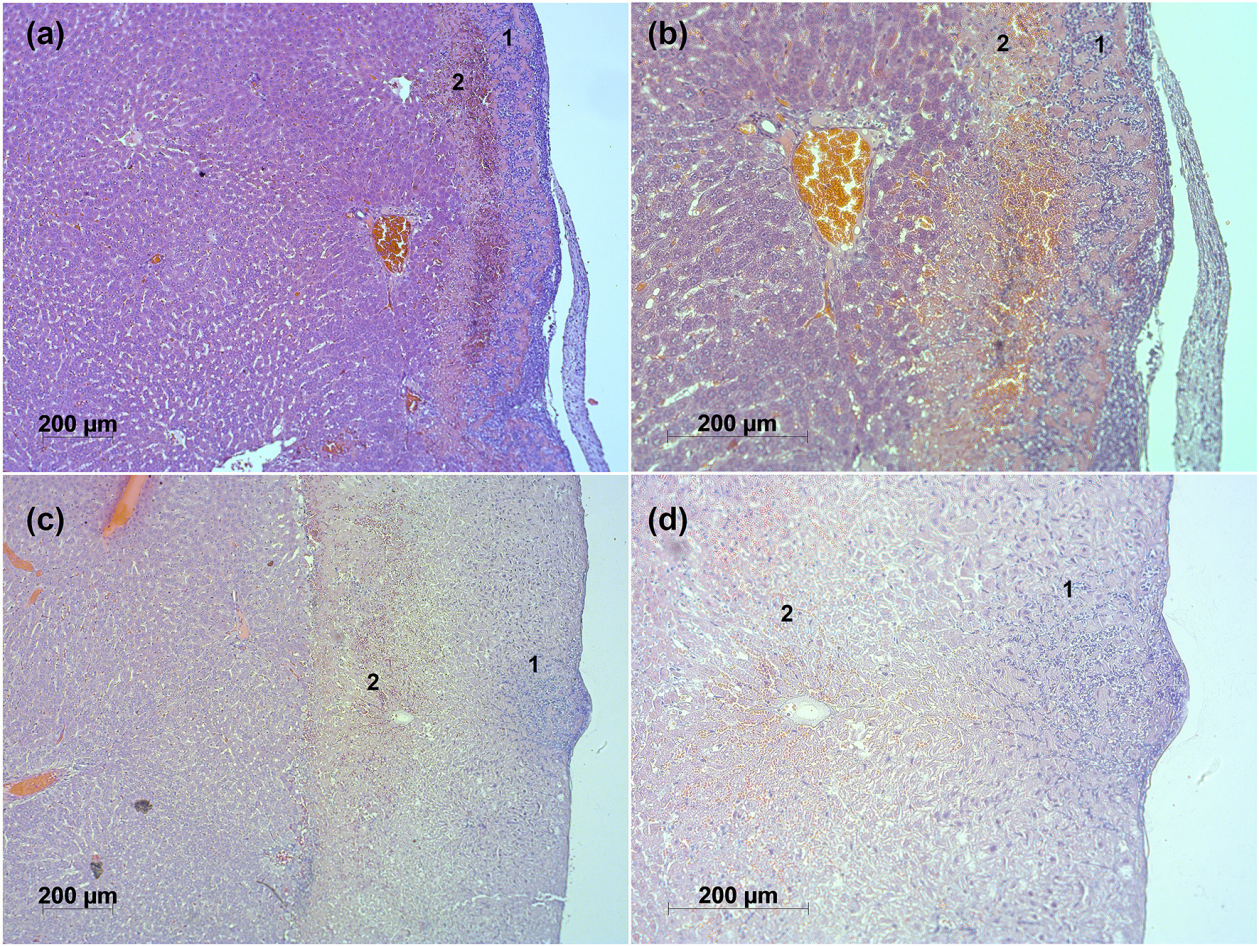
Representative histopathological slides in different magnifications. (a) G4: ES+PS+Light, 5x; (b) G4: ES+PS+Light, 10x; (c) G5: PS+ES+Light, 5x; (d) G5: PS+ES+Light, 10x.

In order to assess the tissue morphology after ES and PDT conjugation, a histopathological analysis was performed. Figs 4 and 5 show representative slides of all experimental groups.

In Fig 4A the histological slide of the control group G1 is shown. A severe damaged layer of carbonized cells is observed. Below this layer, an extensive coagulation necrosis is present. Infiltration of neutrophils in comparison to the native tissue occurs in this group and is more concentrated near the remaining tissue surface. Erythrocyte extravasation (hemorrhage) within the necrotic tissue is also observed. In Fig 4B, a representative histological slide from group G2 is shown. The induced necrosis also presents coagulation characteristics. However, the distribution of neutrophils is uniform. Interstitial hemorrhage was also observed in Fig 4B, although with higher intensity in comparison to Fig 4A. The damage pattern in this group is uniform and different in intensity. Fig 4C shows the pre-photosensitized tissue necrosis when subsequent electrosurgical debulking is applied (control group G3). The extravasation of erythrocytes, coagulation and neutrophil infiltration are present within the necrotic tissue, but in a mild or moderate extent.

The previous histological features persisted in the ES+PDT conjugation groups G4 and G5 (Fig 5), but in different intensity and distribution. Two distinct regions of damage can be observed. Fig 5A and 5B show the damage for the protocol sequence ES+PS+Light (G4). In this situation, the necrosis presents two distinct damage regions, marked as 1 and 2. Neutrophil infiltration, coagulation and hemorrhage occur with different intensities and distributions when comparing both regions. An extra region, formed by cells presenting mild hydropic degeneration, also appears in group G4. Fig 5C and 5D show the histopathological features when the conjugation sequence is changed to PS+ES+Light. The statistical average of the observed histopathological damage scores are organized in Table 2.

**Table 2:** Tissue damage summary.

Experimental Groups	Class of damage		
	Hemorrhage	Neutrophil infiltration	Coagulation
G1	+	+ +	+
G2	+ -	+ +	+
G3	+	+ -	-
G4	+ + +	+ + -	+ -
G5	+ -	+ +	+ +

Mean damage intensity for all experimental groups. The values are labeled as mild (+), moderate (++) or severe (+++). Intermediate values between these are designated using a half cross symbol (-).

**Fig 6:**
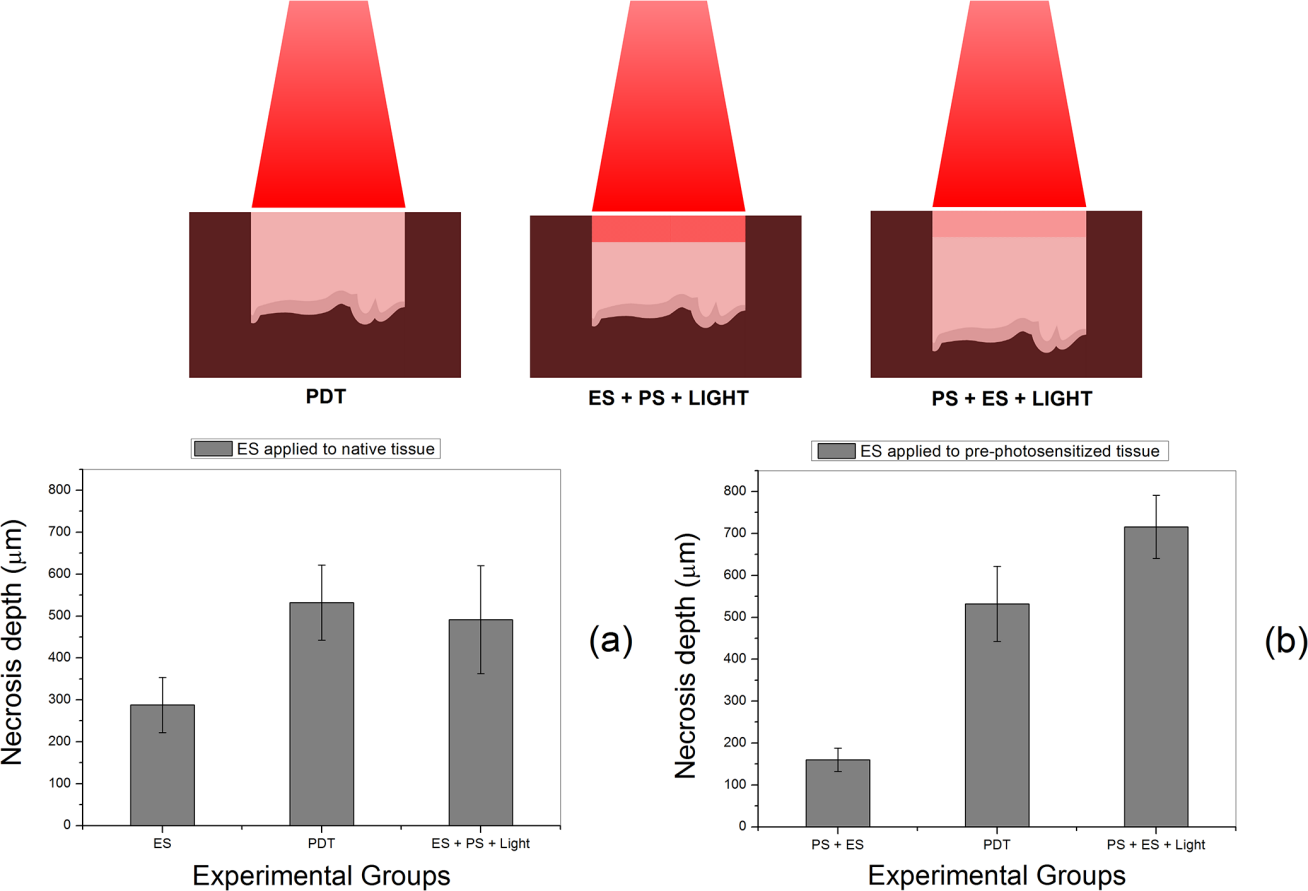
Total necrosis depth analysis. The bars on the graph represent the mean depth of necrosis from groups G1 to G5, from left to right. The error bars are the standard deviations from multiple measurements performed in the histological slides.

Fig 6A and 6B graphically show the calculated necrosis depth of all experimental groups. The ES control group G1 showed a total necrosis depth of (288 ± 66) μm, which decreases when ES is applied to previously photosensitized liver (G3)–(160 ± 28) μm. The PDT treatment, for the applied illumination parameters in the liver, is responsible for (532 ± 90) μm. This last value is comparable to the conjugation group G4, presenting total necrosis depth of (491 ± 129) μm. Finally, the conjugation sequence PS+ES+Light (G5), showed a total necrosis depth of (716 ± 75) μm.

## Discussion

The fluorescence spectra in Figs 2 and 3 represent a quantitative measure of the Photogem availability for PDT in both conjugation sequences. The strong PS fluorescence in Fig 2B would also indicate a minimum PS bleach. Additionally, ES damage caused an increase in the collected optical intensity in Fig 2A. This is explained as part of the fluorescence from the tissue layers is scattered by the tissue itself. Therefore, the ratio of emitted photons that reach the fiber tip increases. Also, the scattering cross section increases for short wavelengths, and the green region (near 540 nm) increases in intensity, contributing to the observed blue shift.

Blood components emit in the red region of the spectrum when excited by a 532 nm laser [31,32]. Additionally, the fluorescence intensity of hepatic tissues decreases after thermal damage [33]. These facts reflect on the observed FWHM. In Figs 2B and 3, the decrease in the FWHM was more intense for photosensitized tissues. This is expected because the liver presents a broader emission band than Photogem. Therefore, when the PS fluorescence predominates, the FWHM decreases. Consequently, the larger FWHM in the ES+PS fluorescence (Fig 3) indicates that the tissue accumulated a smaller concentration of Photogem when ES is applied first. Conversely, the PS concentration is higher for the PS+ES sequence, once the uptake occurs before the ES damage.

The necrosis characteristics are similar to the work of Ferreira et. al. [6], because the same PDT parameters were used. Infiltration of neutrophils is a consequence of inflammatory response, which is an expected outcome of the electrical, thermal (ES) and oxidative (PDT) stresses [34–38]. The interstitial hemorrhage is expected because low diathermy levels were applied. The carbonization and coagulation features observed in the histopathological analysis are a result of the heat produced by electrosurgery. The ES-related damage profile decreases in intensity for deeper tissue layers, indicating the presence of a temperature gradient.

The values on Table 2 are the average damage scores, indicating quantitatively how the applied ES and PDT parameters affect the liver. The coagulation is caused by thermal damage and is mild for groups G1 and G2. The thermal effect of PDT is expected because of the high light absorption from the liver chromophores. The thermal effect of ES stands nowadays as one of the main techniques used to ablate focal hepatocellular carcinomas [39,40]. Consequently, its combined thermal effect with PDT would be clinically beneficial.

The values of Table 2 represent a tendency and cannot be extrapolated to real clinical situations. The neutrophil infiltration is an important parameter to be analyzed in future clinical studies because of its important role in anti-tumor therapies [35–38]. The immediate consequences of electrosurgery, such as bleeding and tissue whitening, are some of the expected features. However, the demonstration that PDT action can be produced below thermal damaged tissue layers is an encouraging scenario for future investigations in tumor models.

Both conjugation groups fulfilled the established criterion of successful outcome, as observed by the different regions of histological damage. In region 1 of group G4, the non-uniform ES-related tissue damage (bleeding, neutrophil infiltration, dehydration and carbonization) is observed. However, region 2 carries the PDT-related damage, similar to Fig 4B, with a uniform distribution of tissue damage, accompanied by a more intense hemorrhage than region 1. In group G5, both regions of damage are observed. In contradiction to the initial hypotheses, the co-occurrence of PDT after electrosurgical debulking was observed in both conjugation sequences.

The fulfillment of the established criterion is also observed in the necrosis depth analysis. Fig 6A shows that the depth of necrosis in group G4 increases from its respective control group (G1). The total depth is equivalent to the PDT necrosis alone. For group G5, the combined necrosis depth increases and is approximately the sum of the depths of both independent damages (G3 + G2). The explanation to such behavior is related to the PDT threshold dose [14]. The tissue modifications induced by ES decreases the PDT damage threshold in region 1, resulting in more photons available to produce photodynamic action in region 2. The expected result would be the sum of the depths of the independent treatments in Fig 6B. However, in Fig 6A, interstitial hemorrhage is present before illumination, causing more light attenuation and decreasing the PDT-related necrosis depth.

The cause for the lower damage profile observed in groups G3 and G5 is unknown. However, a simple theoretical model for heat generation would explain the observed behavior. Thermal damage is a consequence of the Joule effect, which is in turn a consequence of the Ohm’s law of Electromagnetism. It states that heat is generated when electrons flow inside conductors. The caused temperature difference is given by the expression [41]:

$$T-T_{0}=\left(\frac{1}{\sigma \rho c}\right)J^{2}t$$ (1)

The variables *T* and *T*_0_ are the final and initial temperatures respectively; *ρ* is the tissue mass density; *c* is the tissue specific heat and *t* is the time of interaction. By expression (1), a lower temperature rise depends on the square of *J*. Consequently, a decrease on the thermal damage must be caused by a decrease on the current density *J*, or an increase on the electrical conductivity *σ*. The Ohm’s law of Electromagnetism states that these two quantities are directly proportional, and an increase on *σ* causes automatically the rise on *J*. Therefore, the decrease on the thermal damage would be caused by an increase in electrical conductivity. Photosensitizers are organic dyes, which are good conductors, making this assumption consistent. Finally, a decrease in the current density would also mean an increase in the transverse area *A*, through which the electrical current *i* crosses. Thus the electrons would spread more rapidly when penetrating the photosensitized tissue.

The mean values of Table 2 and the decreased damage in pre-photosensitized tissue also indicate that the delivered RF currents did not interact with the PS to produce singlet oxygen. If the flow of electrons caused such effect, the damage to the tissue in group G3 would be more intense than in group G1, and the data show the opposite behavior. It also corroborates with the non-observed PS bleach in the fluorescence analysis. Finally, it is worth mentioning that the RF currents did not produce observable spams of vessels and tissue during the experiments, based on the tested ES parameters [28] and the type of anesthesia administered.

## Conclusion

In conclusion, Photodynamic Therapy can be successfully used as adjuvant to electrosurgery in both of the proposed conjugation sequences, as confirmed by histopathological analysis and necrosis depth measurements. One specific sequence, namely the PS+ES+Light, presented a superior total depth of treatment, which would be more recommended in the clinical ablation of lesions. The important physical quantities during the conjugation, including tissue electrical conductivity, heat diffusion, tissue fluorescence and light penetration, are different when the procedure sequence is altered. The competition between these quantities influences the histological damage score and its distribution. As a consequence, the features observed in the present experiments need to be taken into consideration for clinical studies in which electrosurgery is used as a debulking procedure before Photodynamic Therapy.
